# TGA class II transcription factors are essential to restrict oxidative stress in response to UV-B stress in Arabidopsis

**DOI:** 10.1093/jxb/eraa534

**Published:** 2020-11-14

**Authors:** Ariel Herrera-Vásquez, Alejandro Fonseca, José Manuel Ugalde, Liliana Lamig, Aldo Seguel, Tomás C Moyano, Rodrigo A Gutiérrez, Paula Salinas, Elena A Vidal, Loreto Holuigue

**Affiliations:** 1 Departamento de Genética Molecular y Microbiología, Facultad de Ciencias Biológicas, Pontificia Universidad Católica de Chile, Santiago, Chile; 2 Millennium Institute for Integrative Biology (iBio), Santiago, Chile; 3 FONDAP Center for Genome Regulation, Santiago, Chile; 4 Escuela de Biotecnología, Facultad de Ciencias, Universidad Santo Tomás, Santiago, Chile; 5 Centro de Genómica y Bioinformática, Facultad de Ciencias, Universidad Mayor, Santiago, Chile; 6 Bielefeld University, Germany

**Keywords:** Arabidopsis, glutathione *S*-transferase, GSTU7, photooxidative stress, redox signaling, ROS, TGA class II, TGA2, transcription factor, UV-B stress

## Abstract

Plants possess a robust metabolic network for sensing and controlling reactive oxygen species (ROS) levels upon stress conditions. Evidence shown here supports a role for TGA class II transcription factors as critical regulators of genes controlling ROS levels in the tolerance response to UV-B stress in Arabidopsis. First, *tga256* mutant plants showed reduced capacity to scavenge H_2_O_2_ and restrict oxidative damage in response to UV-B, and also to methylviologen-induced photooxidative stress. The *TGA2* transgene (*tga256/TGA2* plants) complemented these phenotypes. Second, RNAseq followed by clustering and Gene Ontology term analyses indicate that TGA2/5/6 positively control the UV-B-induced expression of a group of genes with oxidoreductase, glutathione transferase, and glucosyltransferase activities, such as members of the glutathione *S*-transferase Tau subfamily (*GSTU*), which encodes peroxide-scavenging enzymes. Accordingly, increased glutathione peroxidase activity triggered by UV-B was impaired in *tga256* mutants. Third, the function of TGA2/5/6 as transcriptional activators of *GSTU* genes in the UV-B response was confirmed for *GSTU7*, *GSTU8*, and *GSTU25*, using quantitative reverse transcription–PCR and ChIP analyses. Fourth, expression of the *GSTU7* transgene complemented the UV-B-susceptible phenotype of *tga256* mutant plants. Together, this evidence indicates that TGA2/5/6 factors are key regulators of the antioxidant/detoxifying response to an abiotic stress such as UV-B light overexposure.

## Teaser

Arabidopsis TGA class II transcription factors are part of a signaling network that controls ROS levels and oxidative damage in the tolerance response to UV-B and photooxidative stress.

## Introduction

Plants are equipped with a diversity of genetic mechanisms to protect themselves, survive, and adapt to stress caused by changes in environmental conditions and by pathogen attacks (Zhu, 2016; Diaz, 2018). UV-B light, the electromagnetic radiation with the highest energy that reaches the earth’s surface from the sun, is one important source of environmental stress for plants, triggering developmental and defense genetic programs (Ulm and Jenkins, 2015; Yin and Ulm, 2017). Overexposure to UV-B radiation, like most stress conditions, induces an increase in the cellular levels of reactive oxygen species (ROS) (Hideg and Vass, 1996; Brosché and Strid, 2003; Frohnmeyer and Staiger, 2003). Particularly under UV-B stress, superoxide accumulation occurs in the apoplast, due to activation of the NADPH oxidases AtRBOHD and AtRBOHF (Kalbina and Strid, 2006), and in the chloroplasts, due to an effect of UV-B on PSII function (Kulandaivelu and Noorudeen, 1983; Larkum *et al.*, 2001). Superoxide is then converted to H_2_O_2_, which is the longest lasting ROS (Apel and Hirt, 2004).

ROS have a dual effect in the defense response: at higher levels they produce oxidative damage of biomolecules such as lipids, proteins, and DNA (Das and Roychoudhury, 2014), while at lower levels they act as signals for the activation of developmental genetic programs and defense responses (Foyer and Noctor, 2013; Considine *et al.*, 2015; Mignolet-Spruyt *et al.*, 2016; Noctor *et al.*, 2018; Fichman and Mittler, 2020). Accordingly, one of the critical challenges for plants, as sessile organisms exposed to stressful environmental changes, is to sense and control ROS levels in order to properly respond to environmental signals, survive, and adapt.

Plant cells possess a robust network of metabolic systems involved in sensing and controlling ROS levels (Foyer and Noctor, 2013; Farooq *et al.*, 2019). These systems use non-enzymatic components, such as the redox buffers glutathione, ascorbate, and NADPH, and enzymatic components represented by a diversity of multifunctional peroxidases, reductases, and dehydrogenases (Foyer and Noctor, 2011). These systems not only scavenge ROS keeping their levels low, but are also part of the cellular redox signaling network that transmits oxidative signals to trigger defense and developmental responses (Noctor *et al.*, 2018). Considering this dual role, Noctor *et al* (2018) proposed to refer to these enzymatic systems as ‘ROS-processing systems’.

The main plant ROS-processing systems are the well-recognized antioxidative enzymes that directly detoxify ROS in different organelles (superoxide dismutase, catalase, and ascorbate peroxidase) and that regenerate reduced forms of redox buffers (monodehydroascorbate reductase, dehydroascorbate reductase, glutathione reductase, and NADPH-thioredoxin reductase) (Noctor *et al.*, 2018). Supporting the robustness of ROS metabolism in plants, ROS-processing systems also include other enzymes encoded by large multigene families, such as peroxiredoxins (PRXs), glutaredoxins (GRXs), thioredoxins (TRXs), glutathione peroxidases (GPXs), glutathione *S*-transferases (GSTs), and class III peroxidases. Interestingly, these thiol-based enzymes (PRX, GRX, and TRX) and peroxidases (GPX and GST) are encoded by genes highly responsive to biotic and abiotic stress, and to a wide variety of stress-associated compounds such as salicylic acid (SA), jasmonic acid (JA), abscisic acid, and H_2_O_2_ (Sappl *et al.*, 2009; Dixon and Edwards, 2010; Meyer *et al.*, 2012; Bela *et al.*, 2015; Choudhury *et al.*, 2017; Veljovic Jovanovic *et al*., 2018; Liebthal *et al.*, 2018; Sylvestre-Gonon *et al.*, 2019). The functional roles, as well as the mechanisms and signaling pathways involved in the activation of most of these genes are still not fully known (Sewelam *et al.*, 2016).

The TGA transcription factors are bZIP proteins that have been involved in plant responses to biotic stress and in developmental processes. The Arabidopsis genome codes for 10 *TGA* members divided into five classes according to their sequence similarities (Jakoby *et al.*, 2002; Gatz, 2013). The TGA class II, that includes *TGA2*, *TGA5*, and *TGA6*, show a redundant function in defense response against pathogens, being described as essential for the systemic acquired resistance (SAR) against biotrophic pathogens (Zhang *et al.*, 2003), and for the defense response against the necrotrophic pathogen *Botrytis cinerea* (Zander *et al.*, 2010, 2014) in Arabidopsis. This is due to their role as gene regulators in the SA-mediated (Johnson *et al.*, 2003; Zhang *et al.*, 2003; Kesarwani *et al.*, 2007; Blanco *et al.*, 2009) and the JA–ethylene-mediated pathways (Zander *et al.*, 2010), respectively. TGA class II factors have been also involved in controlling the expression of genes with detoxification functions in response to SA (Fode *et al.*, 2008; Blanco *et al.*, 2009; Herrera-Vasquez *et al.*, 2015a), cyclopentenone oxylipins (Mueller *et al.*, 2008; Stotz *et al.*, 2013), and xenobiotics such as 2,4-D (2,4-dichlorophenoxyacetic acid) and TIBA (2,3,5-triiodobenzoic acid) (Fode *et al.*, 2008, Huang *et al.*, 2016).

Current evidence supports a role for ROS as signaling molecules, and a functional role for diverse families of ROS-processing enzymes in the antioxidative defense response to stress. However, the transcriptional regulation mechanisms that link ROS signaling with the effector genes of the defense response are poorly understood. Here, we show that TGA2/5/6 transcription factors are critical regulators of ROS-processing responses to abiotic stress. Accordingly, genetic evidence supports a role for class II TGA factors in the control of H_2_O_2_ levels and oxidative damage in the tolerance response to UV-B light and photooxidative stress, as well as in the control of transcription of a group of genes coding for detoxifying and H_2_O_2_-scavenging enzymes such as GSTU. These results indicate that TGA class II factors are key for cellular redox homeostasis, acting as essential regulators of the antioxidative response induced under stress conditions.

## Materials and methods

### Plant material and growth conditions

*Arabidopsis thaliana* wild-type plants and all mutant lines were in the Columbia (Col-0) background. The mutant lines used were *tga2-1/tga5-1/tga6-1* (*tga256*), *tga2-1/tga5-1* (*tga25*), and *tga6-1* (*tga6*) (Zhang *et al.*, 2003), and *tga2-1/tga5-1/tga6-1* complemented with the *pUBQ:TGA2-V5* construct (*tga256*/TGA2 lines #1 and #2, this report) or the *pUBQ:GSTU7-V5* construct (*tga256/GSTU7* lines #1 and #2, this report). Seedlings were grown *in vitro* in 0.5× Murashige and Skoog (MS) medium supplemented with 10 g l^–1^ sucrose and 0.3% Phytagel (Sigma) in a Percival growth chamber (16 h light, 100 µmol m^–2^ s^–1^, 22±2 °C).

### Genetic constructs and plant transformation

The *pUBQ:TGA2-V5* and *pUBQ:GSTU7-V5* constructs were generated using Gateway technology following the manufacturer’s instructions (Invitrogen). The *TGA2* and *GSTU7* coding regions were amplified from cDNA using the primers described in Supplementary Table S3. The purified PCR products were cloned into the pENTR/SD/D-TOPO vector and then recombined into the pB7m34gw vector to express the proteins fused to a V5 tag controlled by the *UBQ10* promoter that allows a moderate gene expression in nearly all tissues of Arabidopsis (Norris *et al.*, 1993; Grefen *et al.*, 2010). *tga256* mutant plants were transformed by the floral dip method using the *Agrobacterium tumefaciens* C58 strain carrying the corresponding vectors. Transgenic seeds were selected in 0.5× MS solid medium supplemented with 15 µg ml^–1^ glufosinate-ammonium. Two stable homozygous transgenic lines for each construct (indicated as #1 and #2) were used for further analyses.

### Plant stress treatments

The protocol for tolerance assays to germinate in SA is described in Zhang *et al.* (2003) and we used 0.2 mM SA (sodium salicylate salt, Sigma-Aldrich, #31493). For SA treatments to evaluate *GRXC9* gene expression, 15-day-old seedlings were floated on 0.5 mM SA (treatment) or 0.5× MS medium as a control, and incubated for the indicated periods of time (Supplementary Fig. S2) in a growth chamber.

**Fig. 1.**
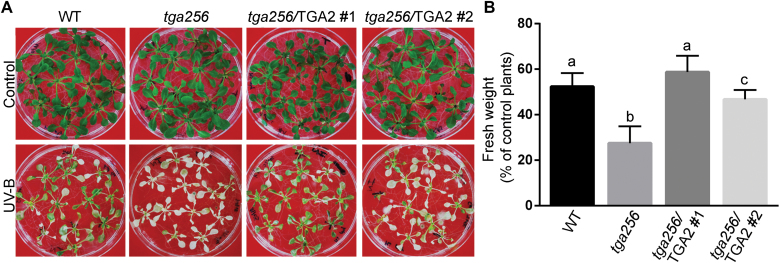
TGA class II factors are essential for tolerance to UV-B stress. Fifteen-day-old seedlings of the indicated genotypes were treated with UV-B radiation for 24 h and then they recovered for 72 h in a growth chamber. Control treatments were performed under the same conditions with a UV-B filter. Pictures (A) and fresh weight measurements (B) were obtained at the end of the recovery period. Fresh weight of rosette tissue from UV-B-treated plants was expressed as a percentage of fresh weight of rosettes from control plants. Bars represent the mean ±SD of at least three independent experiments (20 seedlings per genotype for each experiment). Statistical analysis was performed using ANOVA/Fisher’s LSD test. Different letters denote statistically significant differences at *P*<0.05.

**Fig. 2.**
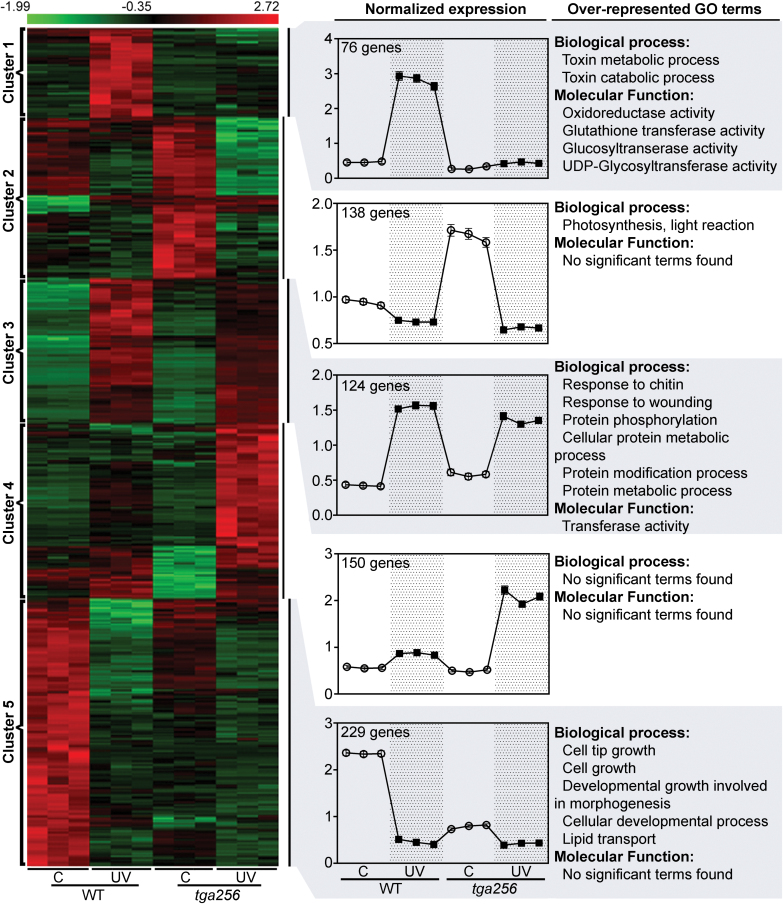
Expression patterns of UV-B-responsive genes regulated by TGA2/5/6 factors. The 717 genes differentially expressed in response to treatment and genotype were grouped hierarchically. The normalized expression data of these genes in three biological replicates from wild-type (WT) and *tga256* mutant plants treated under control (C) or UV-B (UV) conditions are shown. Genes were separated into five different clusters according to their expression profile patterns. The *z*-score of normalized expression for each group of genes under control (C, open circles) and UV-B conditions (UV, filled squares) is shown in the graphs. Error bars represent the SE. The total number of genes is indicated at the upper left side of each graph. The over-represented GO terms were obtained according to the MIPS classification using BioMapstool from VirtualPlant (http://virtualplant.bio.nyu.edu), which compares the number of genes from each GO term in each cluster with the whole Arabidopsis genome. A Fisher exact test with FDR correction was used for the analysis (*P*<0.05).

**Fig. 3.**
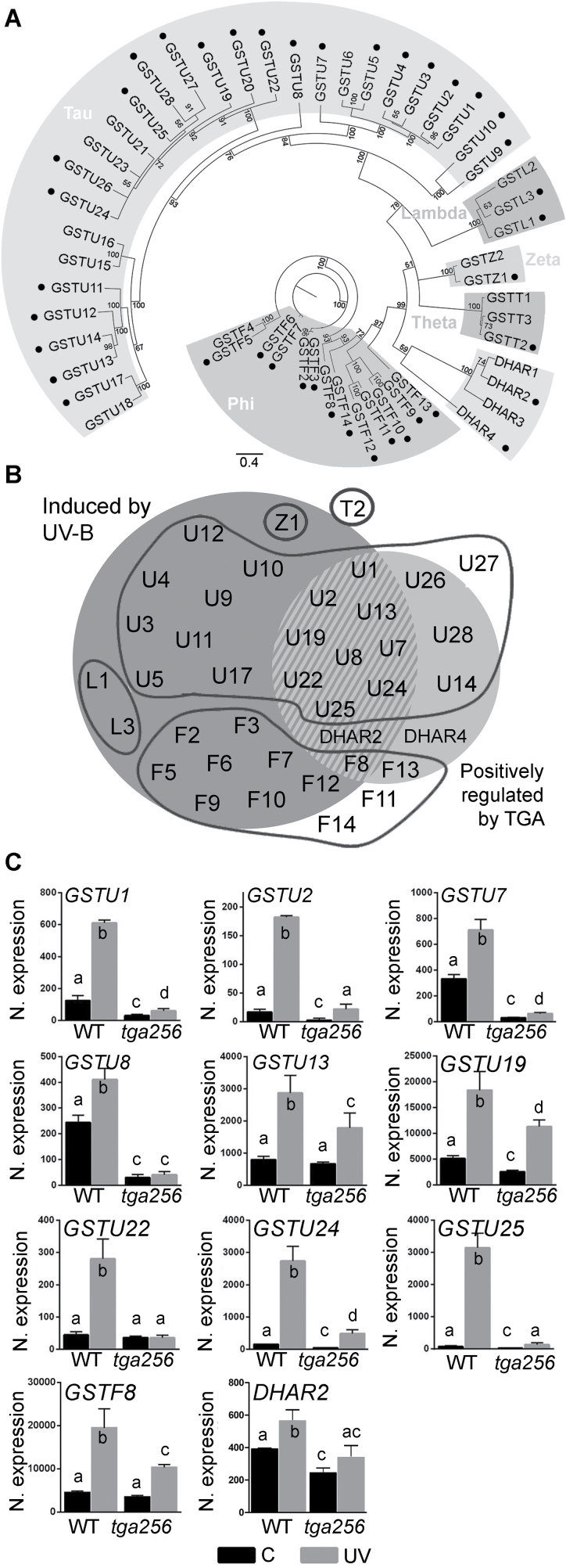
The induction of *GSTU* genes by UV-B stress is impaired in the *tga256* mutant plants. (A) Phylogenetic tree of the GST protein family from Arabidopsis. The protein sequences of Arabidopsis GSTs were aligned using the MUSCLE algorithm, and the phylogenetic tree was calculated using Bayesian statistics with MrBayes with a number of generations of 2×10^6^. Node values indicate posterior probabilities; the scale bar indicates estimated substitutions per site. A graphical representation of the tree was created using Figtree (v1.4.2, http://tree.bio.ed.ac.uk/software/figtree/). The six GST subfamilies described (Moons, 2005) are indicated. The dot-marked *GST* genes were identified in the RNAseq experiment as being regulated by the UV-B treatment, the genotype, or the interaction (TG) (ANOVA, *P*<0.05). (B) Venn diagram of the regulated *GST* genes detected in the RNAseq experiments. *GST* genes, named according to Moons (2005), were grouped taking into consideration their responsiveness to UV-B treatment (dark gray circle) and their positive regulation by TGA class II factors (light gray circle) (ANOVA/Tukey’s HSD test). Continuous lines circle different GST families. (C) Expression levels of *GST* genes that are significantly induced under UV-B treatment and decreased in *tga256* mutant plants (dashed area in B). Results are presented as normalized expression of each *GSTU* gene from RNAseq data in wild-type plants (WT) and in *tga256* triple mutant plants (*tga256*) subjected to UV-B treatment (UV, gray bars) or control conditions (C, black bars). Bars represent the mean ±SD from three biological replicates. Different letters above bars indicate significant differences (ANOVA/Tukey’s HSD test, *P*<0.05).

**Fig. 4.**
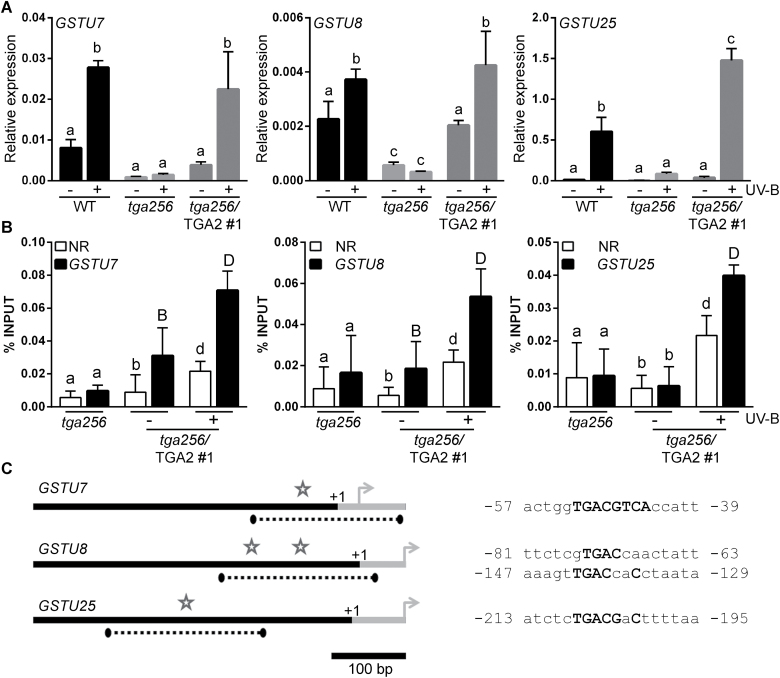
TGA2 controls the UV-B-induced expression of *GSTU7*, *GSTU8*, and *GSTU25* genes via recruitment to their promoters. Wild-type (WT), *tga256* triple mutant (*tga256*), and complemented plants (*tga256*/TGA2 line #1) were irradiated with UV-B for 5 h (+). Untreated plants were used as control (–). (A) The expression of *GSTU7*, *GSTU8*, and *GSTU25* genes was evaluated by RT–qPCR. Data are presented as mean values of *GSTU* gene expression relative to the expression of the housekeeping *YLS8* gene. Error bars represent the SD from three biological replicates (4–5 seedlings each). Different letters above bars indicate significant differences (ANOVA/Fisher’s LSD test, *P*<0.05). (B and C) The *in vivo* binding of TGA2-V5 to the *GSTU7*, *GSTU8*, and *GSTU25* gene promoters was evaluated by ChIP assays. (B) The *in vivo* binding of TGA2-V5 to the *GST* promoters was evaluated in *tga256*/TGA2 #1 complemented plants, using the anti-V5 antibody. *tga256* mutant plants were used as negative controls for immunoprecipitation. The proximal promoter regions of *GSTU7*, *GSTU8*, and *GSTU25* were evaluated by qPCR. The coding region of the *ACTIN2* gene, which does not contain TGA-binding elements, was used as a non-related region (NR). The values of immunoprecipitated DNA samples were expressed as the percentage of a non-immunoprecipitated sample (%INPUT). The differential use of upper- or lowercase letters indicates significant differences between binding of TGA2 to the proximal region of the *GSTU* gene evaluated and the non-related zone in each condition (ANOVA/Fisher’s LSD test, *P*<0.05). (C) Structure of the proximal promoter region of the genes evaluated. Arrows indicate the position of the translation start codon; +1 indicates the transcription start site and the gray bars indicate the transcribed regions. Dotted lines indicate the amplified regions in the qPCR. The stars indicate the position of TGACG motifs in the amplified regions. The bar indicates the length of 100 bp. The sequences of the TGACG motifs are shown (right panel). The numbers indicate distance in base pairs from the transcription start site.

To evaluate tolerance to UV-B irradiation, 15-day-old seedlings grown in plates with solid medium were exposed to UV-B light (0.210 mW cm^–2^) in a chamber equipped with two USHIO UVB F8T5.UB-V, UVP 3400401 fluorescent tubes (λ=306 nm) during 24 h and then recovered for 72 h in the growth chamber under controlled conditions (16 h light, 100 µmol m^–2^ s^–1^, 22±2 °C). As a control, we used seedlings treated in the UV-B chamber and covered with a 320 nm cut-off polyester filter (clear sheet 0.003×20×25'', Grafix® Plastics). Samples were taken at the indicated times during the UV-B treatment or the recovery period to analyze fresh weight (Fig. 1, Fig. 7, Supplementary Fig. S3), H_2_O_2_ levels (Fig. 6), and gene expression by quantitative reverse transcription–PCR (RT–qPCR) (Fig. 4, Supplementary Fig. S4) and by RNAseq (Fig. 2, Fig. 3, Fig. 4). For ion leakage assays, 10-day-old seedlings floated in MilliQ water were exposed to UV-B as indicated above.

To evaluate Arabidopsis lines for tolerance to germinate in methylviologen (MeV; 1,1'-dimethyl-4,4'-bipyridylium chloride), seeds were surface sterilized, spread on solid 0.5× MS medium supplemented with 0.1 µM MeV, stratified at 4 °C for 48 h in the dark, and then germinated and grown under standard controlled conditions. After 15 d, the number of green seedlings compared with the total number of germinated seeds (% survival) was recorded. To detect H_2_O_2_ accumulation after MeV treatment, a 2 µl drop of MeV solution (0, 15, and 30 µM) was placed on the surface of 20 leaves from different 15-day-old plants grown *in vitro*. Plants were then maintained under constant light (100 µmol m^–2^ s^–1^) for 24 h and H_2_O_2_ staining was performed as described below.

### Ion leakage assays

Ion leakage was measured in seedlings subjected to UV-B irradiation for different periods of time. For each sample, three 10-day-old seedlings were floated abaxial side down on a 12-well plate with 2 ml of MilliQ water, and incubated under UV-B as indicated above. The conductivity of the bathing solution was then measured at 22 °C with a conductimeter. The seedlings with the bathing solution were introduced into sealed tubes, and sterilized by autoclaving. The bathing solution was measured again and this value was referred to as 100%. For each sample, ion leakage was expressed as percentage leakage referred to its corresponding 100%.

### Analysis of H_2_O_2_ levels

Accumulation of H_2_O_2_*in situ* after UV-B and MeV treatments was detected using DAB (3,3'-diaminobenzidine, Sigma-Aldrich, #D8001) on whole rosettes of 15-day-old seedlings treated with UV-B following the protocol described in Daudi and O’Brien (2012). In UV-B treatments, 10 seedlings from the wild type, *tga256*, *tga256*/TGA2 #1, and *tga256*/TGA2 #2 genotypes were used. The experiment was repeated three times on different days. ImageJ software (Schneider *et al.*, 2012) was used to define a threshold to distinguish the stained tissue and then to calculate the areas. Finally, the percentage of stained area with respect to the total was calculated for each seedling.

### Immunoblot assays

To evaluate the levels of TGA2-V5 and GSTU7-V5 proteins in Arabidopsis lines expressing the *pUBQ:TGA2-V5* and *pUBQ:GSTU7-V5* constructs, commercial monoclonal anti-V5 antibodies (Invitrogen, #R96025) were used. A 30 µg aliquot of total protein of each sample was separated on 15% SDS–polyacrylamide gels and transferred to Immobilon™ polyvinylidene difluoride (PDVF) membranes (Millipore Co., #IPVH00010). The membrane was blocked for 1 h in phosphate-buffered saline–Tween (PBS-T) with 5% non-fat dried milk at room temperature. To detect V5-tagged proteins, a 1:5000 dilution of the primary anti-V5 antibody (Invitrogen, #R96025) and an anti-mouse horseradish peroxidase-conjugated secondary antibody (1:10 000 dilution, goat anti-mouse, KPL, #5220-0341) were used. The immunocomplexes were visualized using Thermo Scientific Pierce chemiluminiscent Western Blotting Substrate (Pierce, #32109) according to the manufacturer’s instructions.

### RT–qPCR analysis

Total RNA of whole seedlings was obtained from frozen samples using the TRIzol® Reagent (Invitrogen, #15596026). cDNA was synthesized with the ImProm II Kit (Promega). qPCR was performed using the Brilliant III Ultra-Fast SYBR® Green QPCR Master mix reagents (Agilent Technologies, #600882) on an AriaMx real-time PCR system. The expression levels of *GRXC9* (AT1G28480), *GSTU7* (AT2G29420), *GSTU8* (AT3G09270), and *GSTU22* (AT1G78340) were calculated relative to the *YLS8* (AT5G08290) housekeeping gene. The expression of *YLS8* and other described housekeeping genes was analyzed from RNAseq data (Supplementary Fig. S1). Primers used for each gene are listed in Supplementary Table S3.

### Library preparation and RNAseq analysis

Total RNA was obtained from 12 samples (UV-B-treated samples for 5 h and control UV-B-filtered samples)×(wild-type and *tga2/5/6* mutant genotypes)×(three independent biological replicates), using the PureLink RNA Mini kit (Thermo Fischer Scientific, #12183018A) as suggested by the manufacturer, using 200 mg of frozen seedlings/sample. The quality of total RNA isolated for library preparation was determined using capillary electrophoresis on a Fragment Analyzer (Advanced Analytical Technologies, #DNF-471). All samples used for library preparation had an RNA quality number (RQN) of ≥8. Library preparation for RNAseq was performed using the TruSeq® Stranded mRNA Library Prep Kit (Illumina, #RS-122-2102), starting from 2 µg of total RNA and following the manufacturer’s instructions. Library quantitation was performed by qPCR using the Library Quant Kit, Illumina GA (KAPA, #KK4824). The size range of the libraries was determined using the High Sensitivity NGS Kit on the Fragment Analyzer (Advanced Analytical Technologies, #DNF-474). The libraries were sequenced on a HiSeq2500, generating 125 bp paired-end reads. Data quality was assessed with FastQC 0.11.6 (https://www.bioinformatics.babraham.ac.uk/projects/fastqc/), and Trimmomatic v. 0.36 (Bolger *et al.*, 2014) was used to remove low quality reads, using the following settings: two mismatches allowed between adapter and seed sequence, minimum of Q=30 for palindromic alignment between adapter and sequence, minimum of Q=10 for simple alignment between adapter and sequence, removal of leading and trailing sequences if Q<3, sliding window of four bases and removal of sequence if Q<15. The sequences were mapped to the TAIR10 Arabidopsis genome using TopHat2.

Read counts per genomic feature (TAIR10 annotation) were determined for each library using the featureCounts function of the Rsubread R package (McCarthy *et al.*, 2012; Liao *et al.*, 2013). Data were median normalized using the R package EBSeq (Leng and Kendziorski, 2019). A constant value was added to normalized reads to avoid negative values in the log2 transformation (30 percentile value ~10 pseudocounts). The log2-transformed data were subjected to a two-way ANOVA (Chambers *et al.*, 1992), with a false discovery rate (FDR) of 5%. For the ANOVA, we used a model considering the expression of a given gene Y as Yi=β0+β1T+β2G+β3TG+ε, where β0 is the global mean; β1, β2, and β3 are the effects of T, G, and the TG interaction; and the variable ε is the unexplained variance. Significant differences after FDR correction were evaluated with TukeyHSD post-hoc test (*P*<0.05) (Miller, 1981; Yandell, 1997).

### Clusters and Gene Ontology analysis

The data were normalized, transforming the values by using the mean and the SD of the row of the matrix to which the value belongs, using the z-score formula: Value=[(Value)–Mean(Row)]/[Standard deviation(Row)]. Normalized expression data of genes belonging to the TG-regulated group were used to generate a hierarchical clustering of genes using Pearson correlation and the average linkage method using the Multiple experiment Viewer analysis software (Howe *et al.*, 2011). Gene Ontology (GO) analyses for over-representation of Biological processes and Molecular function terms were performed using the BioMaps tool at the VirtualPlant webpage (Katari *et al.*, 2010), utilizing as background the TAIR 10 genome, GO assignments by TAIR/TIGR, Fisher exact test, and *P*<0.05.

### Peroxidase activity assays

Protein extracts were obtained from 200 mg of wild-type, *tga256*, and *tga256*/*TGA2* #1 plants, by grinding the frozen tissue in 200 µl of cold extraction buffer [50 mM phosphate buffer pH 7.6, 150 mM NaCl, 0.2% IGEPAL, 5 mM EDTA, 1× protease inhibitor cocktail (Sigma-Aldrich, #P9599)]. Samples were centrifuged at 16 000 *g* and 4 °C for 20 min, and the supernatant was used for assays. The GPX activity was measured as the velocity of decrease in absorbance_(λ=340)_ in 500 µl of reaction mixture [50 mM phosphate buffer pH 7.0, 1 mM GSH, 10 mM H_2_O_2_, 0.15 mM NADPH, and 5 U of glutathione reductase (Sigma, #G3664)] using 50 µg of protein extract. The activity was calculated as (∆Abs_λ=340_/∆*t*) mg^–1^ of protein on the first 180 s of reaction, while the Pearson correlation factor (*R*^2^) was >0.98. For determination of NADPH concentration, ε _NADPH(λ340)_=6150 mol^–1^ cm^–1^ was used. For each reaction, the spontaneous degradation of NADPH was measured prior to protein extract incorporation into the reaction mixture and was subtracted from the measurements with the protein extract incorporated.

### Chromatin immunoprecipitation assay

ChIP assays were performed and analyzed as described (Saleh *et al.*, 2008), using 3 g of fresh leaf tissue per sample. A 5 µl aliquot of the anti-V5 antibody (Invitrogen, #R96025) was used for immunoprecipitation assays. The concentration of DNA in each sample (input chromatin and chromatin immunoprecipitated with either specific or non-specific antibodies) was quantified by qPCR, using the Stratagene MX3000P® equipment and the Sensimix Plus SYBRGreen Reagents (Quantece, #BIO-83005). As control, we consider the amplification of a non-related DNA region that does not include sequences related to TGA-binding sites (TGA boxes) in the amplicon and neighboring regions (1000 bp upstream and downstream). The amplicon was located in the second exon of the *ACT2* gene, 1494 bp downstream of the transcription start site. Primers used to amplify the promoter regions are listed in Supplementary Table S3.

### Accession numbers

*TGA2* (AT5G06950), *TGA5* (AT5G06960), *TGA6* (AT3G12250), *GRXC9* (AT1G28480), *CHS* (AT5G13930), *YLS8* (AT5G08290), *GSTU7* (AT2G29420), *GSTU8* (AT3G09270), *GSTU25* (AT1G17180), and *ACT2* (AT3G18780).

## Results

### TGA class II factors are essential for tolerance to UV-B stress

To evaluate the role of class II TGAs in the defense response to UV-B stress in Arabidopsis, we used the previously characterized *tga2-1 tga5-1 tga6-1* line (*tga256* mutant plant) that carries the combined deletion of *TGA2* (AT5G06950), *TGA5* (AT5G06960), and *TGA6* (AT3G12250) genes (Zhang *et al.*, 2003). We transformed the *tga256* triple mutant with the *pUBQ:TGA2-V5* gene and selected two complemented lines expressing the V5-tagged TGA2 factor, as detected by immunoblot using an anti-V5 antibody (Supplementary Fig. S2A). In order to set up these genetic tools for the UV-B and photooxidative stress studies, we evaluate the functional expression of the *TGA2* transgene by complementation of previously described phenotypes of the *tga256* mutant in the response to SA (Zhang *et al.*, 2003; Ndamukong *et al.*, 2007; Blanco *et al.*, 2009; Herrera-Vásquez *et al.*, 2015a). Despite some differences in the TGA2-V5 protein accumulation level, in both lines the TGA-V5 expression was enough to recover the tolerance to germinate in MS medium supplemented with 0.2 mM SA (Supplementary Fig. S2B), and the SA-controlled expression of the *GRXC9* gene coding for glutaredoxin C9, which is detected in wild-type plants and abolished in the *tga256* mutant (Supplementary Fig. S2C). These results indicate that the V5-tagged TGA2 factor is functional and its expression in the *tga256* mutant background is sufficient to complement previously reported phenotypes of the mutant in the SA-mediated responses.

To assess involvement of class II TGAs in the defense response against UV-B stress, the tolerance to UV-B treatment was assayed in the *tga256* mutant and in the two *tga256*/TGA2 complemented lines. Seedlings were exposed to stress by UV-B radiation during 24 h followed by a recovery period of 72 h under standard growing conditions. As shown in Fig. 1, plants treated with UV-B show higher chlorosis and reduced plant fresh weight, compared with the control plants. The *tga256* mutant plants were more susceptible than wild-type plants to UV-B treatment, a phenotype that was reverted in the two complemented lines (completely in line #1 and partially in line #2).

To analyze whether TGA2/5/6 are redundant in the defense response to UV-B, as was previously reported for SA-mediated responses (Zhang *et al.*, 2003; Herrera-Vásquez *et al.*, 2015a), we evaluated tolerance to UV-B in the *tga2-1 tga5-1* (*tga25*) double mutant as well as in the single *tga6-1* (*tga6*) mutant (Zhang *et al.*, 2003). As shown in Supplementary Fig. S3, plants from *tga25* and *tga6* mutant lines do not show higher susceptibility to UV-B than wild-type plants. Therefore, the three TGA class II genes must be mutated in order for the plant to develop a susceptible phenotype, indicating that the three genes are redundant in this response, as was previously described for the responses to SA (Zhang *et al.*, 2003).

### Identification of UV-B-regulated genes controlled by TGA class II factors

In order to analyze the role of TGA class II in the genetic response to UV-B, we used RNAseq to analyze global expression profiles in wild-type and *tga256* plants exposed to UV-B light. We exposed 15-day-old seedlings of wild-type and *tga256* genotypes to UV-B for 5 h and then total RNA was isolated from complete seedlings. In parallel, we performed control UV-B treatments in plants covered with a filter.

As a control for the response to UV-B treatment, we performed RT–qPCR to analyze the expression levels of the chalcone synthase gene (*CHS*, AT5G13930), a marker gene for this response (Fuglevand *et al.*, 1996; Jenkins *et al.*, 2001) (Supplementary Fig. S4A). Furthermore, as a control for the *tga256* genotype, we analyzed expression data of the *PR-1* gene, which is not induced by UV-B at 5 h post-treatment, but its expression is elevated in the *tga256* mutant due to basal repression mediated by the TGA2/5/6 factors (Zhang *et al.*, 2003) (Supplementary Fig. S4B).

Differential gene expression analysis was assessed using a two-way ANOVA (*P*<0.01). The resulting numbers of genes that are differentially expressed after UV-B treatment (T), in the different genotypes (G), or as a result of the interaction between treatment and genotype factors (TG) are compared in Supplementary Fig. S5, and listed in Supplementary Table S1 (sheet 1).

We found a group of UV-B-responsive genes regulated by TGA class II factors, whose expression is affected by the treatment and genotype interaction (TG, 717 genes in total; see the list in Supplementary Table S2, sheet 1). These genes were clustered according to their patterns of gene expression using hierarchical clustering. We used a figure of merit analysis to determine the optimal number of different clusters that represent the major patterns of expression of TG genes. As shown in Fig. 2, we found five different clusters for which we analyzed the over-representation of GO terms (Biological process and Molecular function; see the results in Supplementary Table S2, sheet 2). Two of the clusters include genes that are up-regulated by UV-B in wild-type plants and whose UV-B induction is strongly abolished (cluster 1, 76 genes) or diminished (cluster 3, 124 genes) in the *tga256* mutant (Fig. 2). As expected, GO terms associated with defense against stress are over-represented in these two clusters. Another two clusters include genes down-regulated by UV-B in wild-type plants whose expression is affected in the *tga256* mutant only under control conditions, being negatively (cluster 2, 138 genes) or positively (cluster 5, 229 genes) regulated by TGA2/5/6 factors (Fig. 2). GO terms associated with photosynthesis (cluster 2), and growth and developmental processes (cluster 5), were over-represented in these clusters (Fig. 2), providing evidence for a negative influence of stress on plant fitness (Heidel *et al.*, 2004; Kempel *et al.*, 2011; Huot *et al.*, 2014). Finally, one cluster (cluster 4, 150 genes) includes genes that are slightly up-regulated by UV-B in the wild type and more strongly up-regulated by UV-B in *tga256* plants, suggesting a negative UV-B response control by TGA2/5/6. Significant GO terms were not found in this group of genes (Fig. 2).

Looking for genes that could be responsible for the phenotype of higher susceptibility to UV-B observed in the triple *tga256* mutant compared with wild-type plants (Fig. 1), we focused on clusters 1 and 3 (Fig. 2). These clusters include stress defense genes up-regulated by UV-B in a TGA2/5/6-dependent manner. Interestingly, over-representation of GO terms in these clusters, particularly cluster 1, suggests that class II TGAs are required for full activation of antioxidant and detoxifying capacity of the plant after stress. Accordingly, we found enrichment of genes with molecular functions of oxidoreductase and glutathione transferase activity in cluster 1, and also several *GST* genes in cluster 3 (Supplementary Table S2, sheet 1). Moreover, considering all differentially expressed genes (Supplementary Table S1), we found that 39 out of the 53 *GST* genes detected in the RNAseq experiment were regulated by UV-B treatment, genotype, or the interaction (dot-marked in Fig. 3A, included in Fig. 3B and listed in Supplementary Table S2, sheet 2). Thirty of them were induced by the UV-B treatment (dark gray circle in Fig. 3B), and 16 were positively regulated by TGA2/5/6 (light gray circle in Fig. 3B). The intersection considers the 11 *GST* genes that had a decreased expression in UV-B treatments in the *tga256* genotype compared with the wild type (dashed gray in Fig. 3B). Interestingly, nine of these *GST* genes induced by UV-B treatment in a TGA class II-dependent manner belong to the *GST* subfamily Tau (*GSTU*). For these genes, there is a significant reduction in the UV-B-induced expression in *tga256* compared with wild-type plants. This reduction is more dramatic for *GSTU1*, *GSTU2*, *GSTU7*, *GSTU8*, *GSTU22*, *GSTU24*, and *GSTU25*, while it is partial for *GSTU13*, *GSTU19*, *GSTF8*, and *DHAR2* genes, indicating involvement of other factors, in addition to TGA2/5/6, in the activation of these genes (Fig. 3C). For most of the genes (*GSTU1*, *GSTU2*, *GSTU7*, *GSTU8*, *GSTU19*, *GSTU24*, *GSTU25*, and *DHAR2*), basal expression was also decreased in the *tga256* mutant (Fig. 3C; Supplementary Table S1, sheet 2), indicating that TGA2/5/6 are also required for expression of these genes under unstressed conditions. Furthermore, the diversity of transcriptional profiles reflects the versatility of TGA class II as transcriptional regulators of *GSTU* genes, in concert with others factors. For example, a critical role for TGA2/5/6 in the UV-B response was observed for *GSTU8* and *GSTU22* using the *tga256* mutant (where the induction is abolished), and for *GSTU1*, *GSTU24*, and *GSTU25* (where the induction is substantially diminished) (Fig. 3C). In contrast, for *GSTU2*, *GSTU7*, *GSTU13*, *GSTU19*, *GSTF8*, and *DHAR2* genes, the treatment induces the transcript in a similar magnitude in wild-type and *tga256* mutant plants (Fig. 3C), suggesting rather an amplificatory effect of TGA class II factors.

We selected three *GSTU* genes that by RNAseq analysis showed different patterns of dependency on TGA class II factors (*GSTU7*, *GSTU8*, and *GSTU25*) and we confirmed by RT–qPCR that the UV-B-induced expression of these genes, and the basal expression of *GSTU7* and *GSTU8*, had been restored in the *tga256*/TGA2 plants, supporting that TGA2/5/6 are required for basal and UV-B-induced expression of these *GSTU* genes (Fig. 4A). The *in vivo* binding of TGA2 to *GSTU7*, *GSTU8*, and *GSTU25* promoters was evaluated by ChIP assays in UV-B-irradiated and non-irradiated *tga256*/TGA2 plants. Binding was evaluated by enrichment in TGA2-V5 recruitment to the *GST* proximal promoter regions, in comparison with a non-related region, using V5 antibody. Binding of TGA2-V5 to the promoter of these three *GST* genes was detected when the plants were irradiated with UV-B light (Fig. 4B). We also detected binding of TGA2-V5 to *GSTU7* and *GSTU8* promoters under basal conditions, consistent with the expression profile of these genes (Fig. 4A). The basal binding of TGA2/5 to the *GSTU7* promoter was previously reported (Fode *et al.*, 2008). The proximal promoter regions selected for ChIP assays contained one or two TGACG motifs, as shown in Fig. 4C. Together, these results indicate that TGA2/5/6 act as transcriptional activators, not only for the UV-B-induced expression of *GSTU7*, *GSTU8*, and *GSTU25*, but also for the basal expression of *GSTU7* and *GSTU8*.

**Fig. 5.**
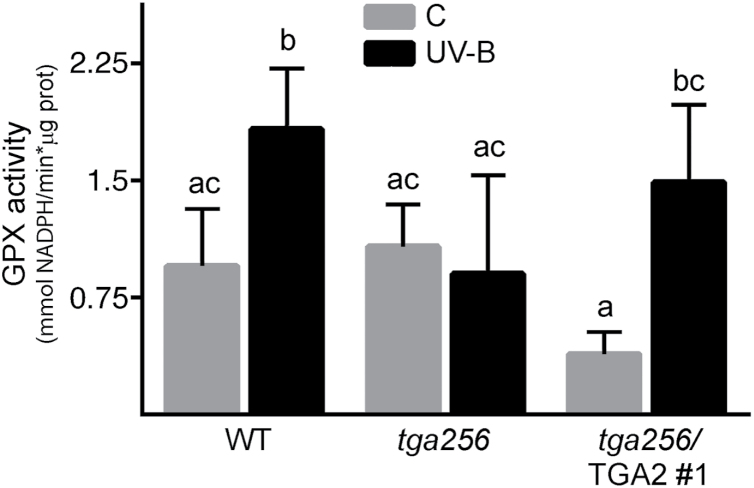
Increase in glutathione peroxidase (GPX) activity upon UV-B treatment in wild-type plants is abolished in *tga256* mutant plants and recovered in *tga256*/TGA2 plants. Wild-type plants (WT), the *tga256* triple mutant (*tga256*), and the complemented line (*tga256*/TGA2 #1) were irradiated with UV-B for 24 h (gray bars) or maintained under UV-B-filtered control conditions (black bars). Total protein extracts were obtained from treated and untreated plants, and the *in vitro* GPX activity was measured. GPX activity is expressed as NADPH consumption considering reaction time and protein concentration (∆mmol NADPH min^–1^ µg of protein^–1^). Values from baselines, given by spontaneous NADPH degradation, were subtracted from enzyme activity values. Data are presented as mean values of GPX activity. Error bars represent the SD from three biological replicates. Each extract sample was prepared using 1 g of seedlings. Different letters above bars indicate significant differences (ANOVA/Fisher’s LSD test, *P*<0.05).

Some GST enzymes possess GPX activity, therefore being part of the H_2_O_2_-scavenging system in plants (Roxas *et al.*, 1997; Cummins *et al.*, 1999; Dixon *et al.*, 2009). In this context, we evaluated whether differences in *GST* gene expression—due to the lack or gain of TGA2/5/6 function—correlate with differences at the level of total GPX activity. Therefore, we assayed GPX activity using H_2_O_2_ as a substrate in total protein extracts from wild-type, *tga256*, and *tga256*/TGA2 plants, under both control and UV-B treatments. In wild-type plants, UV-B irradiation induced an increase in GPX activity, an effect that was lost in the *tga256* mutant plants, and recovered in the TGA2 complemented plants (Fig. 5).

Together, these data indicate that TGA class II factors are essential for UV-B stress-induced expression of a group of *GST* genes and, accordingly, for increased peroxide-scavenging activity under this stress condition.

### TGA class II factors are essential for ROS containment in plants exposed to UV-B light and photooxidative stress

**Fig. 6.**
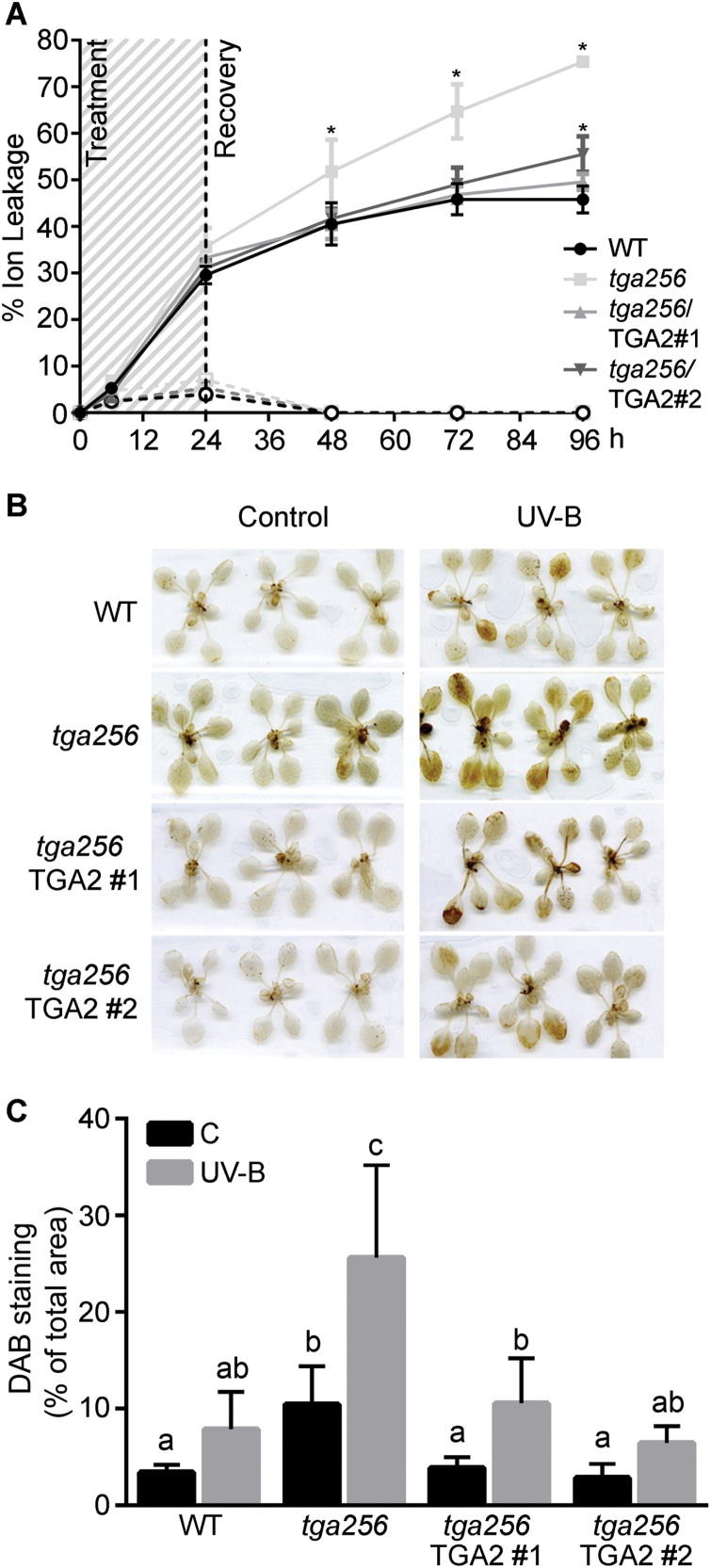
TGA2/5/6 play a critical role in containment of oxidative damage and H_2_O_2_ accumulation in plants treated with UV-B. (A) Oxidative damage was evaluated by measuring ion leakage in 10-day-old seedlings irradiated with UV-B (continued lines) or filtered UV-B as a control condition (segmented lines) at different time points during treatment and recovery periods. Data show mean values ±SD from three replicates. Asterisks represent statistically significant differences with respect to wild-type plants (WT) (two-way ANOVA/Fisher’s LSD test, *P*<0.01). The experiment was repeated three times with similar results. (B) H_2_O_2_ levels were detected using DAB staining in 15-day-old seedlings treated with UV-B radiation for 5 h. (C) The quantification of DAB staining was performed using the ImageJ software, calculating the percentage of stained area with respect to the total rosette area for each seedling. The graph shows the mean value ±SE from 10 WT, *tga256*, *tga256*/TGA2 #1, and *tga256*/TGA2 #2 seedlings irradiated with UV-B light (gray bars) or under control conditions (C, black bars). The experiment was repeated three times with similar results. Different letters above bars indicate significant differences (two-way ANOVA/Fisher’s LSD test *P*<0.01).

Considering that *tga256* mutant plants show a substantial reduction in the UV-B-induced expression of *GST* genes and a concomitant reduction in the peroxide-scavenging activity, we evaluated whether this mutant has an altered redox response to UV-B stress. For this, we measured oxidative damage and H_2_O_2_ levels after UV-B stress treatment in seedlings from the wild type, *tga256* mutant, and *tga256*/TGA2 complemented lines. Oxidative membrane damage was measured by ion leakage assays at different times during and after UV-B treatment. Increased ion leakage in the *tga256* mutant compared with wild-type plants was detected during the recovery period, an effect that was complemented in the TGA2-V5-expressing lines (Fig. 6A). H_2_O_2_ levels accumulated after UV-B treatment were also quantified in plants of all genotypes by *in situ* staining using DAB (Daudi and O’Brien, 2012) (Fig. 6B, C). Increased H_2_O_2_ levels were detected basally and after UV-B treatment in *tga256* mutants compared with wild-type plants, while expression of TGA2-V5 complemented this phenotype, reducing H_2_O_2_ levels to wild-type levels (Fig. 6B-C).

Taken together, these results indicate that expression of TGA class II genes is essential to restrict ROS accumulation under basal and UV-B stress conditions, which correlates with their effect on the levels of *GSTU* gene expression under these conditions.

To further analyze the role of TGA2/5/6 in ROS containment after photooxidative stress, we performed treatments with MeV in the presence of light. Under these conditions, MeV triggers photooxidative stress characterized by increased production of superoxide in the PSI complex (Babbs *et al.*, 1989; Fujii *et al.*, 1990), which in then converted to H_2_O_2_. We germinated seeds from the wild type, *tga256*, and *tga256*/TGA2 lines in 0.5× MS medium supplemented with 0.1 µM MeV and, after 15 d, we recorded the percentage survival (% of germinated seeds that produce green seedlings). In this assay, MeV produced a strong reduction in seedling size in all lines, compared with control seedlings germinated in 0.5× MS medium (Supplementary Fig. S6A). A comparison among lines indicates that mutation of class II TGAs (*tga256* mutant line) produced a significant reduction in the percentage survival compared with the wild type, an effect that was reversed in the *tga256*/TGA2 complemented lines (Supplementary Fig. S6A, B). The higher susceptibility of the *tga256* mutant to germinate in MeV correlates with a lower capacity to restrict H_2_O_2_ accumulation in MeV-treated seedlings (Supplementary Fig. S6C, D). In fact, treatment of leaves from 15-day-old seedlings with a drop of a MeV solution (15 µM and 30 µM) produced the localized accumulation of H_2_O_2_ in the treated tissue, which is less contained and higher in the *tga256* mutant than in the wild type and complemented lines (Supplementary Fig. S6C, D).

Together, these results support a key role for TGA class II factors in the antioxidative response triggered both by UV-B and photooxidative stress conditions, particularly in activating a genetic response able to restrict the ROS accumulation and the oxidative damage in stressed tissues.

### The expression of the *GSTU7* gene complements the UV-B-sensitive phenotype of *tga256* mutant plants

**Fig. 7.**
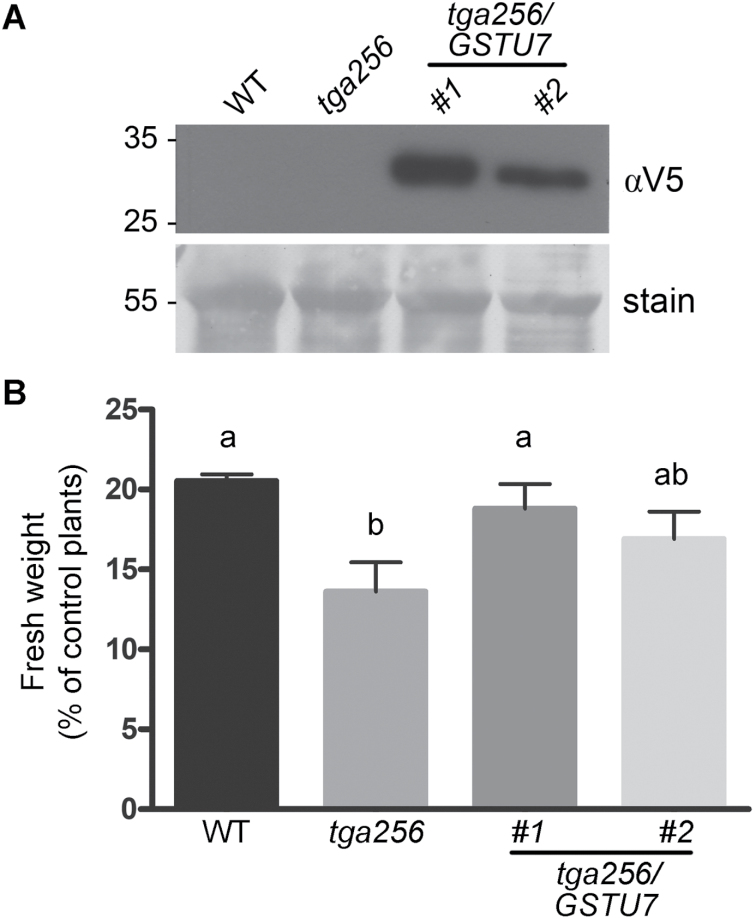
The expression of the *GSTU7* gene complements the UV-B-sensitive phenotype of *tga256* mutant plants. (A) Immunoblot for detection of the GSTU7-V5 protein in two *tga256/pUBQ:GSTU7-V5* complemented lines (*tga256/GSTU7* lines #1 and #2), and in the wild-type (WT) and *tga256* plants as negative controls, using anti-V5 antibody (αV5). Coomassie staining (stain) indicates equivalent protein loading. (B) Fifteen-day-old seedlings of the indicated genotypes were treated with UV-B radiation for 24 h and then recovered for 72 h under normal growth conditions. Fresh weight measurements were obtained at the end of the recovery period. Fresh weight of rosette tissue from UV-B-treated plants was expressed as a percentage of fresh weight of rosettes from control plants. Bars represent the mean ±SD of three independent experiments (20 seedlings per genotype for each experiment). Statistical analysis was performed using ANOVA/Tukey’s HSD test. Different letters denote statistically significant differences at *P*<0.05.

*GST* gene function, particularly that of the *GSTU* subfamily, is over-represented in the group of genes positively controlled by TGA class II transcription factors in response to UV-B stress (Fig. 2). In order to evaluate the involvement of these *GSTU* genes in the tolerance to UV-B stress controlled by TGA class II genes (Figs 4, 5), we selected *GSTU7* as a representative UV-B-induced and TGA2/5/6-controlled gene (Figs 3, 4). We generated transgenic plants that constitutively express *GSTU7* in the *tga256* mutant background. We selected two *tga256/GSTU7* lines (#1 and #2), in which we detected the transgenic GSTU7 protein by immunoblot using anti-V5 antibodies (Fig. 7A), for further analyses.

Consistent with the previous results (Fig. 1), we found a significant decrease in the fresh weight of *tga256* mutant plants compared with wild-type plants after UV-B treatment, indicating increased susceptibility to this type of stress (Fig. 7B). Nevertheless, this phenotype was rescued when the mutant plants constitutively expressed the GSTU7 protein (Fig. 7B), detecting statistical similarity in the fresh weight of both lines and wild-type plants. However, line #2 showed a lower phenotype rescue, which could be attributed to the lower protein levels detected by immunoblot (Fig. 7A). This result suggests that the reduced expression of GSTUs in the *tga256* mutant contributes to the higher susceptibility to UV-B-induced damage.

## Discussion

Here we have reported a critical role for Arabidopsis TGA class II factors (TGA2, TGA5, and TGA6) in the tolerance response to UV-B light and photooxidative stress. Accordingly, we showed genetic evidence (using *tga256* and TGA2-complemented *tga256* plants) supporting that TGA2/5/6 are essential for promoting survival, controlling the H_2_O_2_ level and oxidative damage in response to UV-B light and photooxidative stress induced by MeV. Furthermore, we provided evidence that TGA2/5/6 are essential for UV-B-induced expression of a group of genes coding for detoxifying and ROS-scavenging enzymes, such as GSTU. In particular, we showed that TGA2/5/6 are transcriptional activators of *GSTU* genes in the response to UV-B, as confirmed for *GSTU7*, *GSTU8*, and *GSTU25* genes. On the other hand, we showed that *GSTU* genes play a role in the tolerance response to UV-B stress mediated by TGA2/5/6, as confirmed by the expression of the *GSTU7* transgene in the *tga256* mutant plants. Together, these results are consistent with the idea that TGA2/5/6 play a protective role in oxidative stress tolerance, acting as positive regulators of genes with antioxidant/detoxifying functions, such as *GSTU*.

### GST genes in modulation of H_2_O_2_ levels

The RNAseq and clustering analysis shown here provide genetic support for the susceptibility phenotype of *tga256* mutant plants. Interestingly, many genes coding for peroxide-scavenging enzymes such as GSTUs were detected in the clusters of UV-B-induced genes that are positively regulated by TGA2/5/6 (Figs 2, 3).

GSTUs represent the most numerous and one of the plant-specific subclasses of GSTs in Arabidopsis (Fig. 3A; Moons, 2005). The idea that GSTs play a role in defense responses has been supported by evidence indicating: (i) their transcriptional induction in response to several biotic and abiotic stress conditions and (ii) alterations in tolerance to stress conditions in plants with altered expression levels of some *GST* genes, using genetic tools (Nianiou-Obeidat *et al.*, 2017; Kumar and Trivedi, 2018). In particular, the overexpression of *GSTU* genes has been proved to improve tolerance to oxidative stress produced by H_2_O_2_ (Sharma *et al.*, 2014) and also by MeV (Yu *et al.*, 2003; Xu *et al.*, 2016). Previous reports showed that GST proteins possess GPX activity (Bartling *et al.*, 1993; Cummins *et al.*, 1999; Dixon *et al.*, 2009), and that an increase in GPX activity by overexpression of *GST* genes produces a decrease in ROS accumulation (Cummins *et al.*, 1999; Roxas *et al.*, 2000). Accordingly, we observed that *tga256* mutant plants irradiated with UV-B show a reduction in levels of GPX activity and H_2_O_2_ accumulation, compared with irradiated wild-type plants. The expression of TGA2 in the *tga256* background restores both phenotypes (Figs 5, 6).

### Involvement of TGA class II transcription factors in the stress defense response

TGA class II factors have been previously described as essential for development of the SAR against biotrophic pathogens (Zhang *et al.*, 1999), due to their role as negative and positive transcriptional regulators of SA-induced defense genes, such as *PR-1*, controlled by an NPR1-dependent pathway (Johnson *et al.*, 2003; Zhang *et al.*, 2003; Kesarwani *et al.*, 2007; Pape *et al.*, 2010). NPR1 is a master co-activator that binds TGA factors controlling most of the SA-mediated transcriptional responses (Yan and Dong, 2014). Interestingly, we and others have also reported involvement of TGA2/5/6 factors in other SA-induced pathway(s) that, independently of NPR1, control the expression of groups of genes with detoxification functions (Ndamukong *et al.*, 2007; Fode *et al.*, 2008; Blanco *et al.*, 2009; Herrera-Vasquez *et al.*, 2015a). In this context, the role of TGA2/5/6—and to a lesser extent TGA3—has been further studied for *GRXC9*, a gene induced early by SA that, in contrast to *PR-1*, is induced in the *npr1-1* mutant showing either a partial dependency (Ndamukong *et al.*, 2007) or a complete independency of NPR1 (Blanco *et al.*, 2009; Herrera-Vasquez *et al.*, 2015a). Accordingly, TGA2/5/6 factors are recognized as mediators of SA action under biotic stress, controlling defense gene expression via different mechanisms (Gatz, 2013; Herrera-Vásquez *et al.*, 2015b).

Furthermore, TGA2/5/6 factors have been proposed as a node of crosstalk between the SA- and the JA/ET-mediated pathways (Zander *et al.*, 2012, 2014; Herrera-Vasquez *et al.*, 2015b), due to their role in controlling both the induction by JA/ET and the repression by SA of the *ORA59* gene, which codes for a master regulator of the JA/ET-mediated pathway (Zander *et al.*, 2014). Accordingly, *tga256* mutant plants, in addition to an SAR-deficient phenotype, display increased susceptibility to the necrotrophic pathogen *B. cinerea* (Zander *et al.*, 2014).

Further evidence indicates that TGA2/5/6 are also involved in controlling the expression of groups of genes with detoxification/antioxidant functions in response to cyclopentenone oxylipins (Mueller *et al.*, 2008; Stotz *et al.*, 2013) and to xenobiotics such as 2,4-D and TIBA (Fode *et al.*, 2008, Huang *et al.*, 2016). Accordingly, *tga256* mutant plants are more susceptible than the wild type to germinate in the presence of TIBA (Fode *et al.*, 2008; Huang *et al.*, 2016). In the case of genes induced by oxylipins, a comparison of lists of PPA_1_ (A_1_-phytoprostanes)-induced and SA-induced genes indicated that, even when there is some overlap (19% of PPA_1_-induced genes are also induced by SA), most SA- and PPA_1_-responsive genes are not regulated by both signals (Mueller *et al.*, 2008). These results indicate that genetic responses triggered by oxylipins through TGA2/5/6 factors are not mediated by SA, evidencing the existence of a network of detoxifying/antioxidant genes controlled by different signaling pathways (Mueller *et al.*, 2008).

In this context, the evidence shown here expands our knowledge about the role of TGA class II factors in the stress defense response, placing them as positive regulators of the antioxidative defense response triggered by UV-B light exposure and by photooxidative stress induced by MeV. Accordingly, a clear phenotype of deficiency in the mechanisms that prevent ROS accumulation and oxidative damage was detected in *tga256* mutant plants subjected to both stress conditions. Considering that increases in SA levels have been detected by UV-B stress treatments (Surplus *et al.*, 1998), that there is an interplay between ROS and SA signals (Herrera-Vásquez *et al.*, 2015b), and that SA induces gene expression through TGA2/5/6 factors, we can hypothesize that SA could be mediating the genetic response to UV-B. Nevertheless, evidence indicates that although *GSTU7* can be activated by SA treatment (Fode *et al.*, 2008; Blanco *et al.*, 2009), its induction in response to UV-B and MeV is not dependent on SA (Ugalde *et al.*, 2020, Preprint). We cannot discard the possibility that UV-B- and MeV-induced expression of other *GST* genes could be regulated via SA signaling, or the possibility of crosstalk between the two signaling systems.

In terms of the mechanism, we show that TGA2/5/6 directly regulate the UV-B-induced expression of the *GSTU7*, *GSTU8*, and *GSTU25* genes, as well as the basal expression of *GSTU7* and *GSTU8* (Fig. 4). We detected a correlation between the basal and UV-B-induced transcript levels of these genes (Figs 3C, 4A) and the TGA2 binding to their promoters (Fig. 4C), suggesting that the level of transcriptional activity of these *GSTU* genes can be mediated, at least in part, by the level of TGA2 protein recruited to their promoters. This level of TGA2/5/6 recruitment seems not to be determined by transcriptional activity of *TGA* genes, due to the fact that levels of *TGA2/5/6* gene expression under basal and UV-B treatments were not significantly different (Supplementary Table S1, lanes 4537, 7694, and 7695). Binding of TGA2/5 to the *GSTU7* promoter under basal conditions was previously reported (Fode *et al.*, 2008). For *PR-1* induction triggered by SA, the *PR-1* gene regulation seems also to be mediated by the recruitment of TGA2 (and TGA3) to its promoter (Johnson *et al.*, 2003). In contrast, in the case of *GRXC9* and *ORA59* genes, TGA2 is constitutively bound to the respective promoters under basal or induced conditions, suggesting in these cases a different mechanism for TGA2/5/6 action (Zander *et al.*, 2014; Herrera-Vasquez *et al.*, 2015a).

Supporting the versatility of TGA2/5/6 as transcriptional regulators, here we show that mutation in TGA2/5/6 genes alters in diverse ways the expression profiles of different *GST* genes in response to UV-B. This versatility could be due to the participation of other regulatory or co-regulatory proteins. In fact, the involvement of additional activator(s) in the transcriptional control of several *GSTU* genes induced by UV-B is strongly suggested by the residual expression levels detected in *tga256* mutant plants (Fig. 3C). Furthermore, several co-regulatory proteins have been identified acting as TGA2/5/6-interacting partners at the promoter of different genes, and during different stages of the defense response. This is the case of NPR1 (Johnson *et al.*, 2003), GRXC9 (Ndamukong *et al.*, 2007; Zander *et al.*, 2012; Herrera-Vasquez *et al.*, 2015a), and the SCL14 protein (Fode *et al.*, 2008). Interestingly, SCL14 acts as a positive co-regulator of *GSTU7* expression in response to SA and 2,4-D, which binds to the *GSTU7* promoter through the interaction with TGA class II (Fode *et al.*, 2008). This diversity of regulatory or co-regulatory factors, which interact with TGA2/5/6 factors in different gene promoters, could determine the mechanism by which the TGA factors activate transcription in each case.

Considering that at least two of the co-activators that bind TGA2/5/6 factors (NPR1 and GRXC9) act as redox sensors, we previously proposed TGA2/5/6 as a potential node for redox regulation of defense gene expression (Herrera-Vasquez *et al.*, 2015b). On one hand, NPR1 monomerization, nuclear location, and binding to TGA2/5/6 factors are controlled by the redox state of particular Cys residues (Kinkema *et al.*, 2000; Mou *et al.*, 2003;  Tada *et al.*, 2008). On the other hand, GRXC9, which binds TGA2/5/6 forming part of the transcriptional complex in its own promoter (Herrera-Vásquez *et al.*, 2015a), and possibly in the *ORA59* promoter (Zander *et al.*, 2014), can mediate redox regulation of proteins because of its capacity to catalyze disulfide transitions. Then, the promiscuous and essential role of TGA2/5/6 in the control of genes that respond to the cellular redox state, in association with co-regulatory factors that act as redox sensors, led us to propose TGA2/5/6 as a potential node for redox regulation, particularly in the defense response to stress (Herrera-Vásquez *et al.*, 2015b). In this context, the question of whether TGA class II members perceive the redox signals through redox modification of Cys residues, particularly catalyzed by GRXC9 during its association, is a critical point that still needs to be answered. Expression in the *tga256* mutant background of TGA2 and TGA5 point mutated in the unique potential target site of GRXC9 (Cys186) did not reveal any evidence for this redox modification (Huang *et al.*, 2016; Findling *et al.*, 2018). A further analysis of induction of *GSTU* genes by UV-B stress mediated by TGA2/5/6 factors is required to identify possible factors and cofactors involved in sensing stress signals.

Supporting the participation of TGA2/5/6 in redox control mediated by ROS, among the studies focused on describing *cis*-elements in gene promoters associated with oxidative stress (Garretón *et al.*, 2002; Petrov *et al.*, 2012; Wang *et al.*, 2013), our previous report shows that the TGA-binding motif *as-1* acts as an oxidative stress-responsive element (Garretón *et al.*, 2002).

The evidence shown here provides further support for the idea that TGA class II transcription factors represent a redox regulatory node in stress responses. Thus, TGA2/5/6 not only seem to be responsive to redox signals for controlling activation/repression of different groups of genes, but they also impact on the cellular redox state by controlling the expression of genes responsible for restraining ROS accumulation, and therefore oxidative damage in response to stress.

## Supplementary data

The following supplementary data are available at *JXB* online.

Fig. S1. Expression analysis of the described housekeeping genes upon UV-B treatments.

Fig. S2. Expression of TGA2 complements the *tga256* mutant phenotype.

Fig. S3. TGA class II are redundant in the response to UV-B.

Fig. S4. *CHS* and *PR-1* expression levels in wild-type and *tga256* mutant plants in response to UV-B treatment.

Fig. S5. Global expression analysis of wild-type and *tga256* triple mutant plants in response to UV-B treatment.

Fig. S6. TGA2/5/6 factors are essential for tolerance and H_2_O_2_ control in response to photooxidative stress.

Table S1. List of genes regulated by treatment, genotype, or the interaction (Sheet 1). List of *GST* genes (Sheet 2).

Table S2. UV-B-responsive genes regulated by TGA2/5/6 factors. List of genes differentially regulated by the interaction between treatment and genotype (Sheet 1). Gene Ontology (GO) term enrichment analysis detail (Sheet 2).

Table S3. Primers used for cloning, ChIP and RT-qPCR assays.

eraa534_suppl_Supplementary_Figures

eraa534_suppl_Supplementary_Table_S1

eraa534_suppl_Supplementary_Table_S2

eraa534_suppl_Supplementary_Table_S3

## Data Availability

The large-scale datasets can be found in the Sequence Read Archive (SRA) submission: SUB2037222 Transcriptomics analysis of the response of wild-type and tga2/5/6 Arabidopsis seedlings to UV-B, Oct 26 ‘16 https://www.ncbi.nlm.nih.gov/sra/?term=PRJNA352413.
