# Supplementary material for: TGA class II transcription factors are essential to restrict oxidative stress in response to UV-B stress in Arabidopsis
Source: J Exp Bot. 2020 Nov 14;72(5):1891–905. doi: 10.1093/jxb/eraa534 (PMC7921300; doi:10.1093/jxb/eraa534)
Supplement: eraa534_suppl_Supplementary_Figures [file eraa534_suppl_supplementary_figures.pdf]

A

B

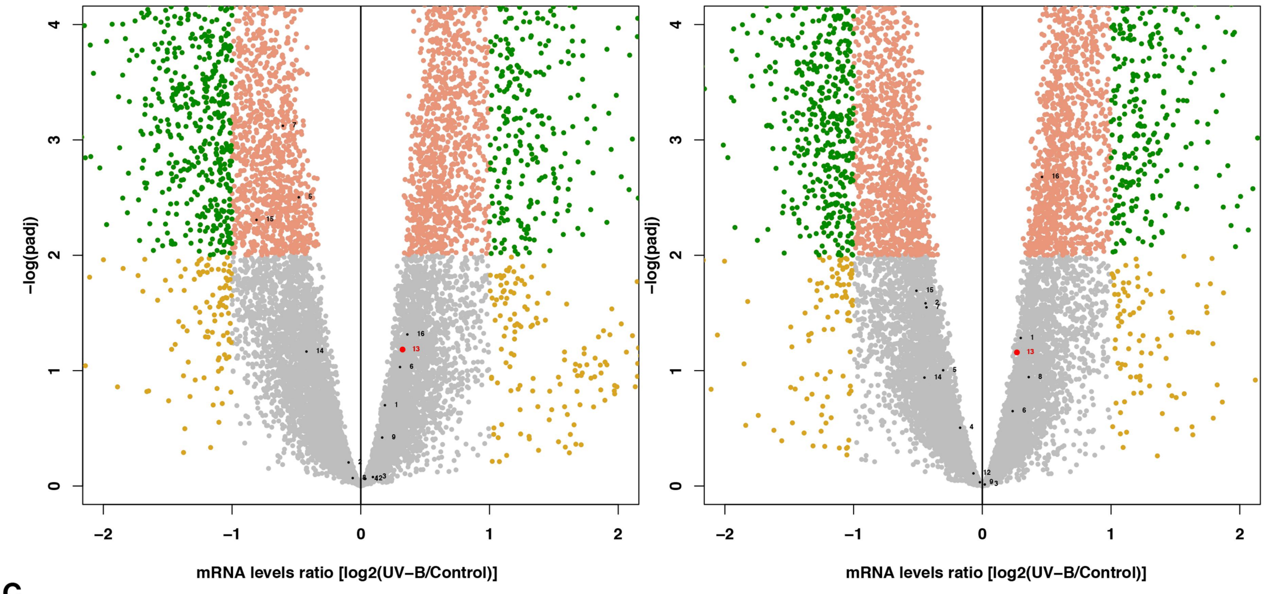

C

	UV/ Control Wt						UV/ Control tga256	
baseMean	log2FoldChange	padj	symbol	Graph ID	Agi	baseMean	log2FoldChange	padj
1173.9	0.19	2.0.E-01	PP2AA3	1	AT1G13320	967.2	0.30	5.2.E-02
20809.0	-0.09	6.2.E-01	GAPC2	2	AT1G13440	13371.4	-0.44	2.6.E-02
66.7	0.09	8.4.E-01	RPF3	3	AT1G62930	49.1	0.02	9.7.E-01
790.2	0.03	8.7.E-01	AT2G28390	4	AT2G28390	538.0	-0.17	3.1.E-01
499.1	-0.48	3.1.E-03	AT2G32170	5	AT2G32170	390.6	-0.30	9.9.E-02
473.9	0.30	9.3.E-02	PTB1	6	AT3G01150	332.0	0.24	2.2.E-01
10012.0	-0.60	7.5.E-04	ACT2	7	AT3G18780	7446.6	-0.43	2.8.E-02
504.5	-0.06	8.5.E-01	UPL7	8	AT3G53090	431.1	0.36	1.1.E-01
712.6	0.17	3.8.E-01	AT4G26410	9	AT4G26410	479.8	-0.02	9.3.E-01
1928.9	0.94	1.4.E-11	UBC9	10	AT4G27960	1284.0	0.87	4.5.E-08
774.7	-0.85	6.8.E-08	AT4G33380	11	AT4G33380	465.6	-1.19	4.9.E-10
408.6	0.04	8.6.E-01	AT4G34270	12	AT4G34270	331.4	-0.07	7.8.E-01
1669.9	0.32	6.6.E-02	YLS8	13	AT5G08290	1290.2	0.27	7.0.E-02
212.4	-0.42	6.8.E-02	AT5G12240	14	AT5G12240	126.0	-0.45	1.1.E-01
7429.6	-0.81	4.9.E-03	TUB4	15	AT5G44340	4382.3	-0.51	2.0.E-02
1487.4	0.36	4.9.E-02	AT5G46630	16	AT5G46630	1127.4	0.46	2.1.E-03

D

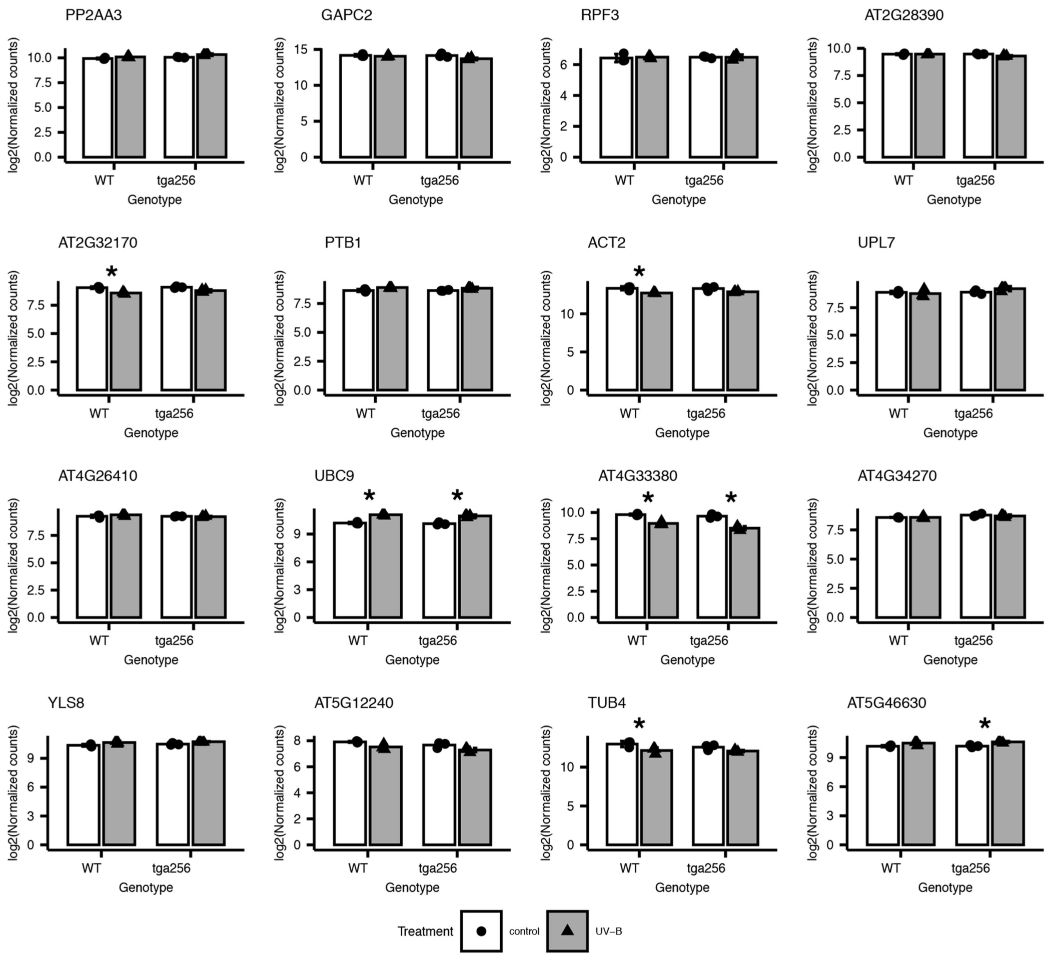

Fig. S1. Expression analysis of described housekeeping genes upon UV-B treatments. RNA levels for different housekeeping genes were evaluated in Wild type (WT) and *tga256* mutant plants by comparing UV-B with control conditions in RNAseq data. Sixteen normalizer genes were selected considering some of the most frequently used (*ACT2*, *TUB4*, and *GAPC2*) (Czechowski et al., 2005; Dekkers et al., 2011) and a set of stably expressed genes shared in different lists for Arabidopsis normalizers (Czechowski et al., 2005; Hong et al., 2010; Wang et al., 2014). **A-B**. Volcano plots showing transcriptomic data for UV-B response for WT (**A**) and *tga256* mutant (**B**) plants. The fold change (UV-B/Control) and the respective FDR-adjusted p-value (padj) was calculated using DESeq2 package (Love et al., 2014) and graphed in logarithmic scale. The dots represent each gene, and they are colored according to the significance of the changes (green: |log2FoldChange|>1, adjusted p-value <0.01; pink: |log2FoldChange|<1, adjusted p-value<0.01; yellow: |log2FoldChange|>1, adjusted p-value >0.01; gray: |log2FoldChange|<1, adjusted p-value >0.01), thus the gray dots represent those genes whose transcript accumulation does not vary significantly in response to the UV-B treatment. The evaluated housekeeping genes are indicated with black dots and numbers. *YLS8* is highlighted in red. **C**. DESeq2 output summary for selected normalizer genes in response to UV-B treatments on WT and *tga256* mutant plants. **D.** Bar graphs showing the normalized counts of the selected housekeeping genes in WT and *tga256* mutant plants. RNA levels for three independent biological replicates are indicated for control (White, circles), and UV-B (Gray, triangles) conditions. Asterisks (*) indicate significant differences (adjusted p-value<0.01) of RNA levels between treatments for each genotype.

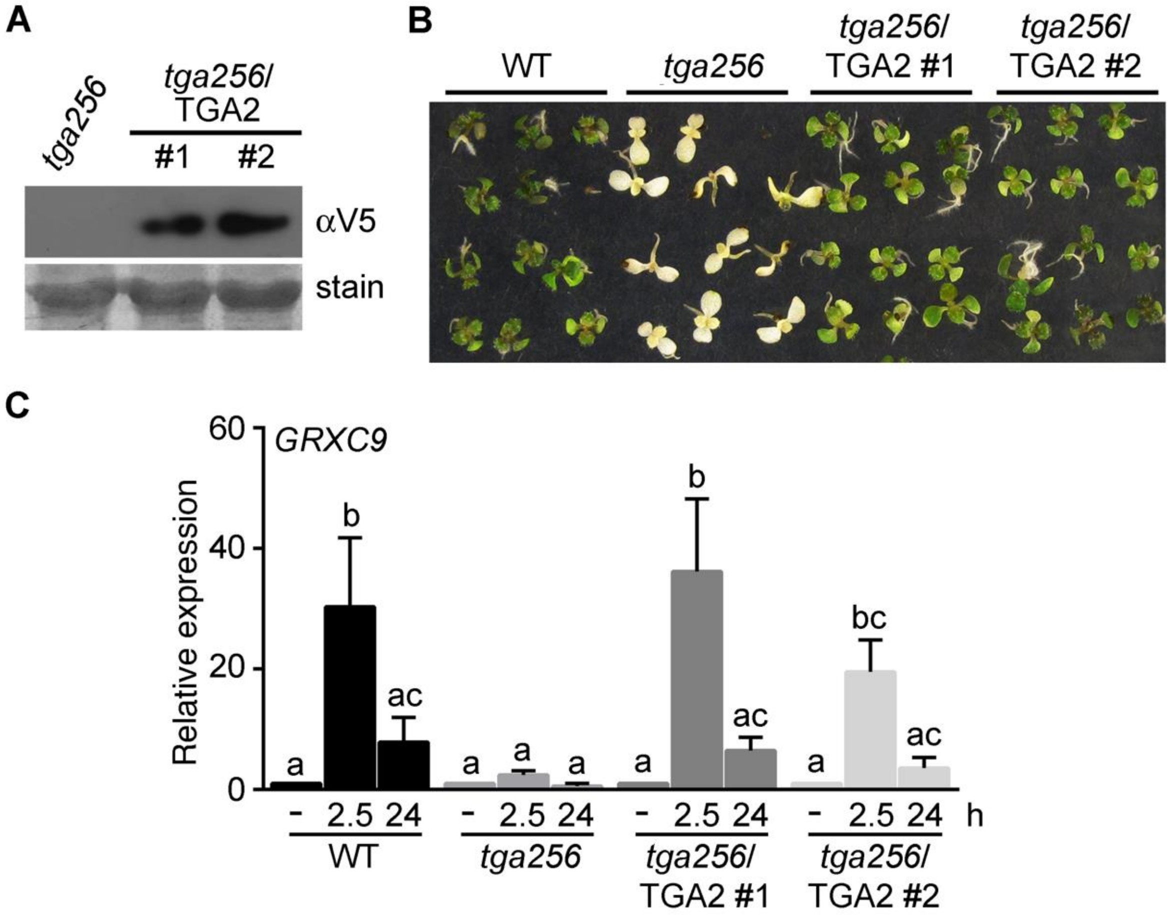

Fig. S2. Expression of TGA2 complements the *tga256* mutant phenotype. (A) Immunoblot for detection of the TGA2-V5 protein in two *tga256/pUBQ:TGA2-V5* complemented lines (*tga256/*TGA2 lines #1 and #2), and in the *tga2-1 tga5-1 tga6-1* mutant (*tga256*) as a negative control, using anti-V5 antibody (αV5). Coomassie staining (stain) indicates equivalent protein loading. **(B)** Assay of tolerance to germinate in SA. Seeds from wild type (WT), *tga256* mutant and the two *tga256*/TGA2-complemented lines were plated on 0.5X MS medium supplemented with 0.2 mM SA. The figure shows survival after 15 days. **(C)** Expression analysis of the *GRXC9* gene evaluated by RT-qPCR in 15-day-old seedlings (WT, *tga256*, and the two *tga256*/TGA2 complemented lines), under basal conditions (-) and after treatment with SA 0.5 mM for 2.5 and 24 h. *GRXC9* relative expression was calculated by normalizing *GRXC9* transcript levels to transcript levels of the housekeeping gene *YLS8* (AT5G08290), and to the WT basal condition. Bars represent the mean ± standard error values from at least three biological replicates. Statistical analysis was performed using ANOVA/Fisher's LSD test. Different letters denote statistically significant differences at $p<0.05$.

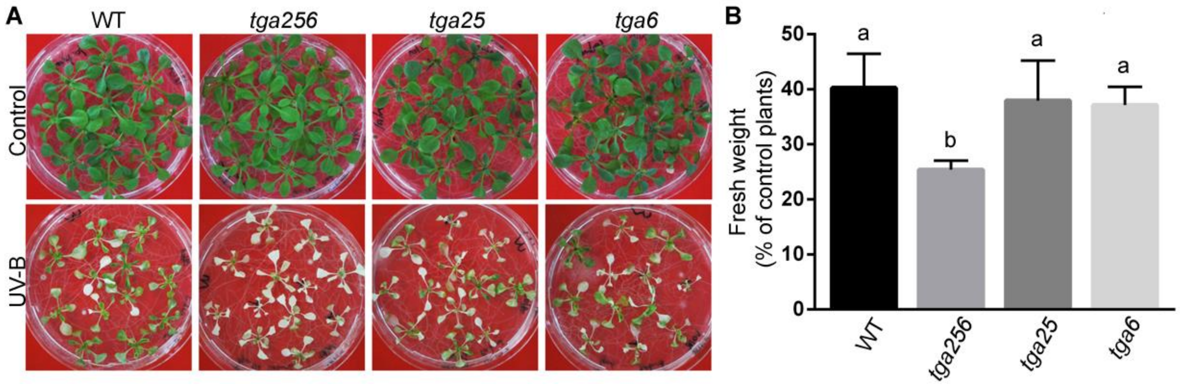

Fig. S3. TGA class II are redundant in the response to UV-B. Fifteen day-old seedlings of WT, *tga256*, *tga2-1 tga5-1* (*tga25*) and *tga6-1 (tga6)* mutant plants were treated with UV-B radiation for 24 h and then they recovered for 72 h in a growth chamber. Control treatments were performed under the same conditions with a UV-B filter. Pictures **(A)** and fresh weight measurements **(B)** were obtained at the end of the recovery period. Fresh weight of rosette tissue from UV-B treated plants was expressed as percentage of fresh weight of rosettes from control plants. Bars represent the mean ± standard deviation of at least 3 independent experiments (20 seedlings per genotype for each experiment). Statistical analysis was performed using ANOVA/Fisher's LSD test. Different letters denote statistically significant differences at $p<0.05$.

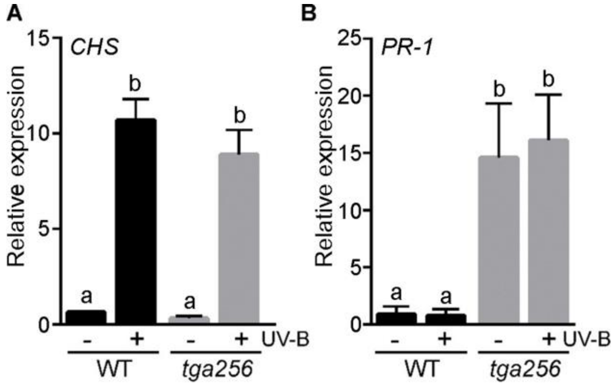

Fig. S4. *CHS* and *PR-1* expression levels in wild type and *tga256* mutant plants in response to UV-B treatment. Expression levels of *CHS* (A) and *PR-1* (B) genes were measured by RT-qPCR in 15-day-old seedlings from wild type (WT, black bars) and tga256 mutant plants (gray bars) exposed to UV-B light during 5 hours (+). As a control, we used seedlings covered with a cellulose acetate polyester filter (-). Relative expression was calculated by normalizing gene transcript levels to transcript levels of the housekeeping gene *YLS8* (AT5G08290). Error bars represent the mean ± SD from 3 replicates. Different letters above bars indicate significant differences (ANOVA/Fisher's LSD test, $p<0.05$)

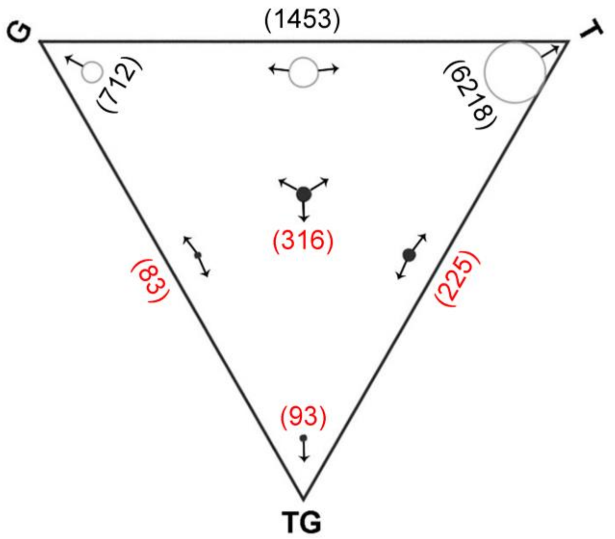

Fig. S5. Global expression analysis of wild type and *tga256* triple mutant plants in response to UV-B treatment. RNAseq data analyzed using two-way ANOVA (p<0.01) is represented using the Sungear tool and show the number of genes that are differentially expressed (Poultney et al., 2007). The triangle shows the factors at the vertices: UV-B treatment (T), genotype (G) and the interaction between UV-B treatment and genotype (TG). The circles inside the triangle represent the number of genes (in parentheses) controlled by the different factors, as indicated by the arrows around the circles. The size of each circle is proportional to the number of genes associated with that circle. White circles represent the genes differentially regulated by genotype or treatment alone; black circles and red numbers represent the 717 genes differentially regulated by the interaction between treatment and genotype (Table S1). The list of genes included in the diagram is presented in Table S2.

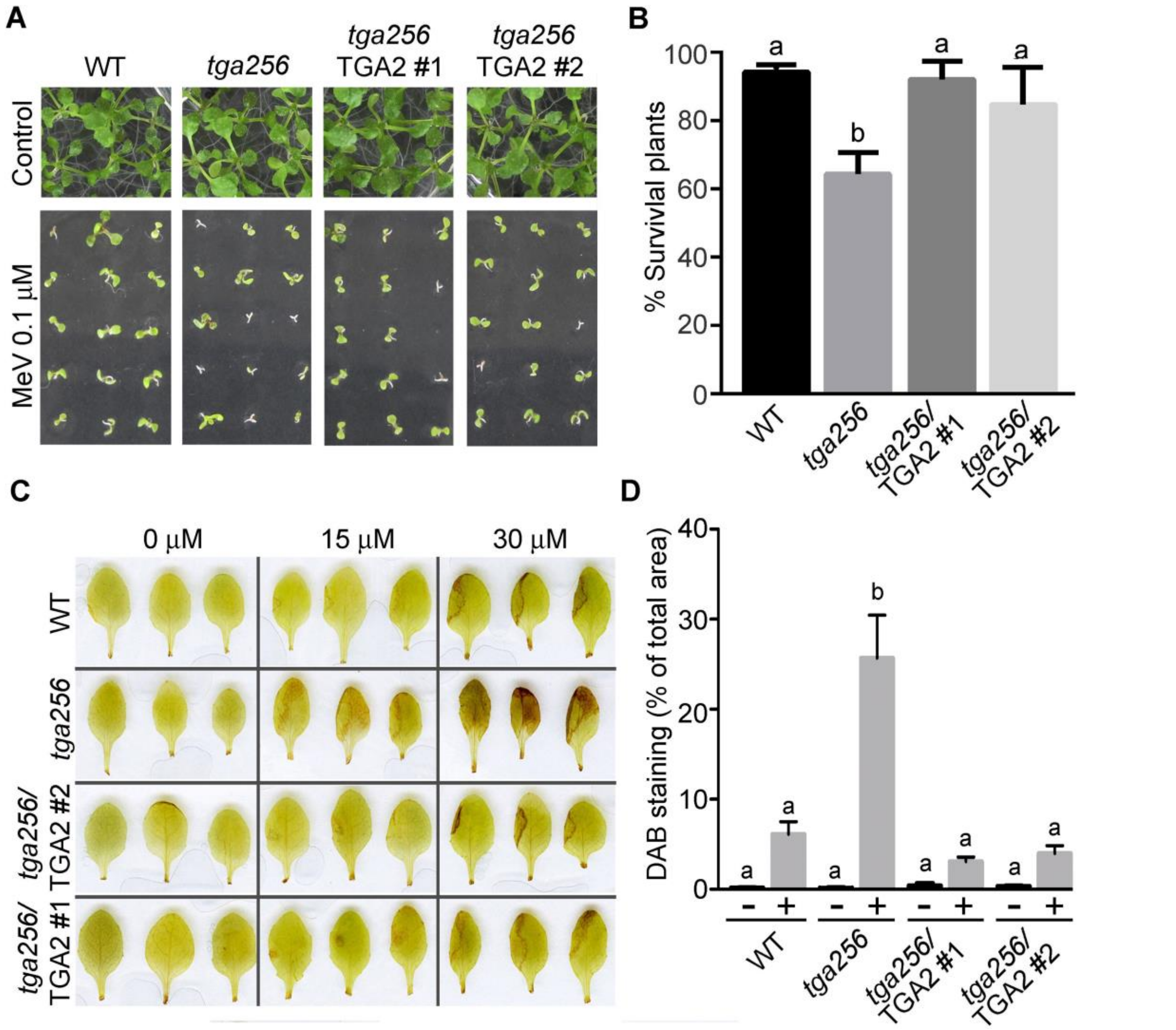

Fig. S6. TGA2/5/6 factors are essential for tolerance and H_2O_2 control in response to photo oxidative stress. (A-B) Seeds from wild type plants (WT), the *tga256* mutant and the two *tga256*/TGA2 complemented lines were germinated in ½ MS medium alone (control) or in ½ MS medium supplemented with 0.1 µM MeV. After 15 days, pictures of seedlings were taken **(A)** and the % plant survival (% of green seedlings respect to the total germinated seeds) was recorded **(B).** Data show mean values ± standard deviation of 4 independent experiments (104 seeds each). Letters represent statistical differences between genotypes (one-way ANOVA/Fisher's LSD test, $p<0.01$). **(C)** Leaves from 15-day-old WT, *tga256* mutant and two *tga256*/TGA2 complemented lines seedlings were treated with a 2 µL drop of MeV (0, 15 and 30 µM) and incubated under constant light for 24 hours. ROS accumulation in the treated leaves was detected by DAB staining (1 mg/ml during 4 hours). The figure shows representative images of leaves from different treated plants (n=20). This experiment was repeated three times with similar results. **(D)** The quantification of DAB staining was performed using the ImageJ software, calculating the percentage of stained area respect to the total leaf area. Graph shows the mean value ± standard error from at least 11 leafs on the wild type (WT), *tga256*, *tga256*/TGA2 #1 and *tga256*/TGA2 #2 genotypes treated with 30µM of MeV (+, gray bars) and untreated conditions (-, black bars). The experiment was repeated three times with similar results. Different letters above bars indicate significant differences (two-way ANOVA/tukey test $p<0.05$).

Table S1. List of genes regulated by treatment, genotype, or the interaction (Sheet 1). List of *GST* genes (Sheet 2).

Table S2. UV-B responsive genes regulated by TGA2/5/6 factors. List of genes differentially regulated by the interaction between treatment and genotype (Sheet 1). Gene Ontology (GO) term-enrichment analysis detail (Sheet 2).

Table S3. Primers used for cloning, ChIP and RT-qPCR assays.

References

Czechowski T, Stitt M, Altmann T, Udvardi MK, Scheible WR. (2005) Genome-Wide Identification and Testing of Superior Reference Genes for Transcript Normalization in Arabidopsis. Plant Physiology **139:**5-17.

Dekkers, B. J. W., Willems, L., Bassel, G. W., van Bolderen-Veldkamp, R. P. (Marieke), Ligterink, W., Hilhorst, H. W. M., & Bentsink, L. (2011). Identification of Reference Genes for RT–qPCR Expression Analysis in Arabidopsis and Tomato Seeds. Plant and Cell Physiology, **53(1)**, 28–37.

Hong SM, Bahn SC, Lyu A., Jung HS, Ahn JH (2010) Identification and Testing of Superior Reference Genes for a Starting Pool of Transcript Normalization in Arabidopsis. Plant and Cell Physiology, **51:** 1694–1706.

Love, M. I., Huber, W., & Anders, S. (2014). Moderated estimation of fold change and dispersion for RNA-seq data with DESeq2. Genome biology, **15**(12):550.

Poultney CS, Gutierrez RA, Katari MS, Gifford ML, Paley WB, Coruzzi GM, Shasha DE (2007) Sungear: interactive visualization and functional analysis of genomic datasets. Bioinformatics **23:** 259-261

Wang H, Wang J, Jiang J, Chen S, Guan Z, Liao Y, Chen F (2014) Reference genes for normalizing transcription in diploid and tetraploid Arabidopsis. Science Reports **4:**6781.
