# Supplementary material for: TGA class II transcription factors are essential to restrict oxidative stress in response to UV-B stress in Arabidopsis
Source: J Exp Bot. 2020 Nov 14;72(5):1891–905. doi: 10.1093/jxb/eraa534 (PMC7921300; doi:10.1093/jxb/eraa534)
Supplement: eraa534_suppl_Supplementary_Table_S3 [file eraa534_suppl_supplementary_table_s3.pdf]

Table S2. Primers used for cloning, ChIP and RT-qPCR assay

Gene/allele	AGI	Use	Direction	Sequence
GRXC9	AT1G28480	RT-qPCR	Forward	5’- CACTCCAAGTCCAAGAAGCAG - 3’
			Reverse	5’- AGAGAGTTCGGATGGTGGTG - 3’
YLS8	AT5G08290	RT-qPCR	Forward	5'- TTACTGTTTCGGTTGTTCTCCATTT -3’
			Reverse	5'- CACTGAATCATGTTCGAAGCAAGT -3’
PR-1	AT2G14610	RT-qPCR	Forward	5'- ACACGTGCAATGGAGTTTGTGG -3'
			Reverse	5'- TTGGCACATCCGAGTCTCACTG -3'
CHS	AT5G13930	RT-qPCR	Forward	5'- TTCCGCATCACCAACAGTGAAC -3'
			Reverse	5'- CGCACATGCGCTTGAACTTCTC -3'
GSTU7	AT2G29420	ChIP	Forward	5' - TCTTCCGATGTGGGACAAAGTG - 3'
			Reverse	5' - ACTCGCCCACATTCCCAAAAG - 3'
		RT-qPCR	Forward	5' - TGTGACGGCGATGAAAGTTGTG - 3'
			Reverse	5' - AATCTCTCGTCGCTTCAACCACAG - 3'
		Cloning	Forward	5' - CACCATGGCGGAGAGATCAAATTCA - 3'
			Reverse	5' - AGCAGATTTGATATTGAG - 3'
GSTU8	AT3G09270	ChIP	Forward	5' - ACAGGCCTTCAACCACTACC - 3'
			Reverse	5' - TCCCTTTGTGTGTGTGTGTT - 3'
		RT-qPCR	Forward	5' - CACAAAGGGAAAGCCAAACCGG - 3'
			Reverse	5' - CGTTTACGTGCTCTTCTTGGTTCA - 3'
GSTU25	AT1G17180	ChIP	Forward	5'-TTTTGGTAATGTATAACCCCTTGA -3'
			Reverse	5'-TGATTAAACATTTTCTTTTCATTAGC -3'
		RT-qPCR	Forward	5'- TCAGCATTGAAGCCGAGTGTCC -3'
			Reverse	5'- TAGCCACACTCTCTCTCTCCACAC -3'
ACT2	AT3G18780	ChIP	Forward	5'- CTTGCACCAAGCAGCATGAA - 3'
			Reverse	5' - CCGATCCAGACACTGTACTTCCTT - 3'
TGA2	AT5G06950	Cloning	Forward	5'- caccATGGCTGATACCAGTCC - 3'
			Reverse	5' - CTCTCTGGGTCGAGCAAG - 3'
